# Psychometric properties of the intercultural effectiveness scale in a sample of in-service Chilean EFL teachers

**DOI:** 10.3389/fpsyg.2026.1734563

**Published:** 2026-02-17

**Authors:** Maura Klenner-Loebel, José Luis Gálvez-Nieto, Juan Carlos Beltrán Véliz, Carmen Gloria Ormazabal Astudillo, Ignacio Norambuena-Paredes

**Affiliations:** 1 Departamento de Lenguas, Literatura y Comunicación, Universidad de La Frontera, Temuco, Chile; 2 Departamento de Trabajo Social, Universidad de La Frontera, Temuco, Chile; 3 Núcleo Científico Tecnológico en Ciencias Sociales y Humanidades, Universidad de La Frontera, Temuco, Chile; 4 Escuela de Pedagogía, Universidad de La Frontera, Temuco, Chile

**Keywords:** EFL, intercultural communication competence, intercultural effectiveness, reliability, teachers, validity

## Abstract

This study aims to validate the adaptation of the Intercultural Effectiveness Scale (IES) to the Chilean Spanish language context within a sample of 393 in-service Chilean EFL teachers. Intercultural effectiveness, a critical dimension of Intercultural Communication Competence (ICC), is essential for promoting culturally responsive pedagogy in diverse classrooms. The IES, comprising six factors—Behavioral Flexibility, Interaction Relaxation, Interactant Respect, Message Skills, Identity Maintenance, and Interaction Management—was analyzed using confirmatory factor analysis (CFA). The results confirmed the six-factor structure of the scale, with adequate fit indices. McDonald’s *ω* coefficients ranged from 0.608 to 0.723, indicating moderate reliability; however, values close to 0.60 reflect limited precision and suggest room for future improvement. Convergent validity was supported through correlations with the Multicultural Awareness Scale (MAS), particularly the Implicit Culture Awareness factor. Although positive correlations were identified, the Message Skills factor demonstrated non-significant correlations, suggesting potential areas for pedagogical improvement. This study highlights the relevance of IES as a tool for enhancing professional development programs that aim to foster ICC in teachers. The findings of this study have implications for curriculum design, intercultural training, and educational policies, particularly in culturally diverse contexts. Future research should focus on the longitudinal impacts of training on teachers’ intercultural effectiveness and on the predictive relationships between ICC and student learning outcomes.

## 1 Introduction

The relation between intercultural dialogue and teaching English as a Foreign Language (EFL) is very complex. EFL teachers are expected to develop high levels of Intercultural Communication Competence (ICC), with a focus on intercultural interaction (Chen, 2014), as well as on intercultural pedagogical competences (Byram, 2020), to foster language learning and connect individuals from diverse cultural backgrounds. ICC involves skills individuals use based on their own culture, relying on appropriate and effective behaviors to communicate effectively with others situated in different sociocultural contexts (Sanhueza Henríquez et al., 2012). Furthermore, ICC consists of affective, behavioral, and cognitive knowledge and skills that allow individuals to develop appropriate and positive behaviors in specific social and cultural contexts, intending to contribute to the development of efficient communication (Vilà, 2014) These components have been defined as the constructs of the Triangular Model of ICC (Chen, 2017) (intercultural sensitivity, intercultural awareness and intercultural effectiveness).

The development of ICC by EFL teachers contributes to addressing various aspects involved in intercultural interactions in English. For example, EFL teachers often work with students from different cultural backgrounds. Understanding students’ cultural characteristics helps teachers create a more inclusive classroom environment (Byram, 2020). In this regard, the EFL teacher should appropriately address the differences that arise in the classroom and, at the same time, respond effectively to problems or situations to bridge cultural gaps (Byram, 2000). ICC also facilitates teacher-student relationships, fosters trust and support, and enhances students’ motivation to learn the language. Students who feel appreciated are more likely to participate actively and take more risks in language learning (Brown, 2007).

The ICC also plays a crucial role in developing other competencies. Learning EFL is not merely learning the language but becoming prepared to function effectively in a globalized world. Teachers who adopt an ICC approach help students develop global citizenship competencies, which are essential for interacting effectively with people from diverse cultures (Deardorff, 2006). This approach also helps students develop an open mindset, curiosity, and empathy toward the foreign. Tolerance and adaptability to what is different are essential qualities for both language learning and social interaction. Developing this type of understanding toward cultural differences is crucial to engaging with others across cultural lines (Bennett, 1993).

The Chilean Ministry of Education (MINEDUC) acknowledges the importance of educating EFL teachers in developing ICC. In 2021, the Ministry of Education introduced a set of standards for English teacher training curricula. The Standards for English Teaching Programs (CPEIP, 2021) include Disciplinary Standard E, which focuses on culture and ICC. This standard emphasizes that teaching EFL in Chile should foster students’ integration into a multicultural world, promoting language as a tool for cultural exchange and mutual respect. Standard E also provides descriptors that address both disciplinary knowledge and didactics. In terms of knowledge, teachers must analyze cultural content in texts and genres. For didactics, they should design lessons that build intercultural skills, mediate between cultures, and develop assessments and activities that respect cultural diversity.

In this context, the availability of valid instruments to assess the development of ICC’s constructs among EFL teachers is of paramount relevance. Intercultural Effectiveness, as a construct of ICC (Portalla and Chen, 2010), becomes an essential aspect of EFL teaching, as teachers need to develop specific behaviors that will prompt ICC in their students. Thus, the objective of this study was to validate the Intercultural Effectiveness Scale (IES) in a sample of in-service EFL teachers. This study set two hypotheses: (H1) the scores of the IES will meet adequate levels of validity and reliability in the sample of in-service Chilean EFL teachers, and (H2) the scores of the six factors of the IES will present positive and statistically significant correlations with the three factors of the MAS scale.

### 1.1 ICC models

There is still some theoretical discussion regarding the nature of ICC. Several key issues surrounding the ICC still need to be addressed, primarily due to the challenges posed by technological advancements and globalization (Chen, 2017). In general terms, ICC refers to the ability to move from one’s own culture to another and to communicate effectively and appropriately with people of different languages and cultures (Huang, 2021). At least two theoretical frameworks have sought to conceptualise ICC, including Byram’s (2020) Intercultural Communicative Competence Model, which integrates attitudes (such as openness and curiosity), knowledge (of social groups and cultural practices), skills of interpreting and relating, skills of discovery and interaction, and a strong ethical dimension, which includes responsibility and engagement in social justice; and Chen’s (2014) Triangular Model of ICC.

The Triangular Model of ICC offers a more manageable approach to operationalizing the constructs of the ICC. Although this model does not measure itercultural pedagogical competencies, it provides a sound framework for understanding individuals’ social disposition toward engaging effectively in intercultural interactions. The model highlights three core components: affective, cognitive, and behavioral. Each of these elements contributes to an individual’s overall competence. These components relate to three constructs: intercultural sensitivity, intercultural awareness, and intercultural effectiveness, respectively.

Intercultural Sensitivity refers to the emotional aspects of ICC, particularly an individual’s motivation to engage in intercultural interactions. It encompasses traits such as empathy, openness, and cultural sensitivity. Research has examined intercultural sensitivity in educational contexts, particularly in language-learning environments. For instance, Fritz et al. (2001) found a strong correlation between intercultural sensitivity and successful cross-cultural adaptation among international students. In the field of English as a Foreign Language (EFL), Karabinar and Guler (2013) used the Intercultural Sensitivity Scale (Starosta and Chen, 2010) to assess pre-service English teachers in Turkey. They found that higher levels of intercultural sensitivity were associated with more positive attitudes toward teaching in multicultural classrooms. More recently, Klenner et al. (2021) examined the factor structure of the Intercultural Sensitivity Scale (ISS) in a sample of Chilean university students and found that the original five-factor model proposed by Chen and Starosta (2000) remained stable in terms of dimensions and theoretical coverage. The study highlights the importance of culturally validating measurement tools and suggests that intercultural sensitivity may be conceptualized differently across cultural settings.

Intercultural Awareness involves an individual’s knowledge and empathetic capacity to understand their own and others’ cultures (Chen and Starosta, 1996), including cultural beliefs, values, norms, and worldviews. Understanding these aspects allows individuals to interpret culturally influenced messages accurately and communicate without significant misunderstandings. This allows intercultural awareness to develop and be enriched by the subject’s perception and understanding of their own culture and of other broader cultures (Cabrera and Barreiro, 2021). The Multicultural Awareness Scale (MAS) assesses individuals’ awareness of cultural diversity, sensitivity to cultural issues, and understanding of systemic inequalities. Studies have demonstrated the importance of intercultural awareness in educational and professional settings. The original validation study conducted among Malaysian undergraduate students demonstrated strong psychometric properties, with the MAS showing high internal consistency (Cronbach’s alpha = 0.89) and construct validity confirmed through exploratory factor analysis, revealing four dimensions: self-awareness, cultural awareness, interaction awareness, and similarity awareness (Rozaimie et al., 2011). Further research by Rozaimie and Ali (2014) used the MAS to investigate the relationship between multicultural awareness and intercultural relations in Malaysia’s multiracial society. Their findings supported the scale’s predictive validity, indicating that higher multicultural awareness was associated with more positive intercultural interactions and reduced psychological discomfort in diverse settings. In another study conducted by Rozaimie et al. (2017) in a sample of students from education and administration fields, a final reliability coefficient of 0.81 was obtained for the MAS. This value indicates that the 10 items of the MAS are reliable for assessing people with diverse ethnic origins’ awareness of cultural differences. Additionally, Yakar et al. (2018) applied the MAS to a healthcare context, evaluating intercultural communication competence among nurses. Their study confirmed the scale’s reliability in a professional setting (Cronbach’s alpha = 0.87) and emphasized the importance of multicultural awareness in enhancing patient care. Collectively, these studies highlight the MAS as a reliable and valid instrument for assessing multicultural awareness across diverse cultural and professional contexts.

Intercultural Effectiveness/Adroitness focuses on the ability to translate both affective and cognitive components into appropriate and effective behaviors during intercultural interactions. It encompasses verbal and non-verbal communication skills, as well as adaptability and flexibility in responding to the cultural expectations of various interaction contexts (Chen and Starosta, 1996). This involves showing sensitivity to cultural diversity by providing specific attention effectively (Yilmaz et al., 2020). The following section elaborates on this construct, which is the core of this study.

### 1.2 Intercultural effectiveness

Intercultural Effectiveness/Adroitness is a particularly relevant aspect of ICC that EFL teachers need to develop. By being interculturally effective, EFL teachers enact the most appropriate behaviors to prompt ICC in their students. These behaviors can be seen in actions such as modeling interaction strategies for intercultural dialogue, considering not only the language but also the context in which it is used (Portalla and Chen, 2010), and implementing didactic strategies and classroom dynamics to prompt real-life communication with people from different cultural backgrounds (Byram, 2020), among others. In this regard, intercultural effectiveness for a teacher involves power dynamics within the educational process, leading to the reproduction and transformation of a culture (Bernstein, 1990). EFL teachers contribute to students’ broader development as global citizens by teaching intercultural effectiveness. This helps students not only master English but also become more culturally aware and effective communicators in an interconnected world (Byram et al., 2001).

Portalla and Chen (2010) developed the Intercultural Effectiveness Scale (IES) to measure this construct. Their study evaluated the IES among 204 undergraduate students enrolled in an introductory communication course in the northeastern United States. The final version of the IES comprises six factors and 20 items, with an overall reliability as measured by the Cronbach’s alpha coefficient of 0.85. The results demonstrated that students who scored well on the IES exhibited flexible cultural behaviors, an ability to distinguish appropriate cultural actions, and effective adaptation to various intercultural situations.

The IES has since been used in educational contexts, particularly in higher education. For example, Bates and Rehal (2017) utilized the IES to evaluate the outcomes of cross-cultural exploration and skill development in short-term undergraduate travel programs. Specifically, the IES was implemented in an international service-learning program in Romania, involving 13 students and two program leaders. Participants completed the IES assessment both before and after the program. The intervention considered three critical dimensions: continuous learning, interpersonal engagement, and resilience. Key findings revealed that the intervention helped students recognize their strengths and areas for improvement prior to traveling abroad. It also highlighted areas of growth, stability, or decline in their intercultural effectiveness. Furthermore, the IES provided program leaders with insights into the cross-cultural strengths and challenges of individual students and the group as a whole. Results demonstrated that cultural immersion and time abroad significantly contributed to student learning and development. Overall, the findings suggest that the IES is an effective tool for evaluating students’ intercultural skills and overall intercultural development.

The IES has been widely validated within the Turkish context. Arslan et al. (2015) validated the IES with 352 Turkish university students, focusing on linguistic adaptation, and confirmed the original factor structure of Portalla and Chen’s (2010) model among 204 Turkish nurses. Yilmaz et al. (2020) further validated the IES among 165 undergraduate nursing students at two universities in Turkey. They adapted the original 20-item scale to a 15-item, three-factor structure through linguistic and content validation. The adapted scale demonstrated sufficient internal consistency. Collectively, these studies underscore the reliability and validity of the IES in other language-speaking samples, thereby supporting its use as a robust tool for assessing intercultural effectiveness across diverse contexts.

Research has consistently demonstrated a strong correlation between the IES and scales measuring the other two constructs of ICC: Intercultural Sensitivity (IS) and Intercultural Awareness (IA). Portalla and Chen (2010) identified a significant correlation between the Intercultural Sensitivity Scale (ISS) and the IES (*r* = 0.74, *p* < 0.01), indicating that individuals with heightened intercultural sensitivity are more proficient at recognizing appropriate behaviors in intercultural interactions. Gungor et al. (2021) explored the connection between intercultural effectiveness, intercultural awareness, and xenophobia using the IES, the Multicultural Awareness Scale (MAS) (Rozaimie et al., 2011) and the Xenophobia Scale (van der Veer et al., 2011), respectively. The study involved 257 nursing students and 341 students from vocational health service programs. Findings showed that female students scored significantly higher in intercultural effectiveness, while male students performed better in intercultural awareness. Moreover, a weak negative correlation was identified between the total mean scores of the Xenophobia Scale and the Intercultural Effectiveness Scale (*r* = −0.182, *p* < 0.001). Additionally, a weak positive correlation was observed between the total mean score of the Intercultural Awareness Scale and the mean score of the Xenophobia Scale (*r* = 0.113, *p* < 0.05). The study concludes that incorporating intercultural effectiveness and sensitivity into healthcare programs’ curricula is essential for mitigating xenophobic biases. These results raise questions about the importance of this construct in other professions that involve working with people, such as the education field. In the Chilean context, Beltrán-Véliz et al. (2024) observed statistically significant positive correlations between the IES and ISS’s interaction confidence factor among 584 pre-service teachers, supporting theoretical expectations behavioral flexibility (*r* = 0. 336: *p* < 0.01), interaction relaxation (*r* = 0.661: *p* < 0.01), interactant respect (*r* = 0.387: *p* < 0.01), message skills (*r* = 0.204: *p* < 0.01), identity maintenance (*r* = 0.319: *p* < 0.01), and interaction management (*r* = 0.478: *p* < 0.01).

Considering the importance of intercultural effectiveness in the development of ICC and its pertinence to the Chilean TEFL context, it becomes relevant to have valid instruments to measure this construct, which would allow for collecting valuable data regarding EFL teachers’ levels of intercultural effectiveness. This information will aid in decision-making for pre-service and in-service teacher education.

## 2 Materials and methods

### 2.1 Participants

The data collection method was a non-probabilistic/purposive sampling approach (Kelly, 2011). A total of 393 English teachers participated, ranging in age from 22 to 69 years (mean = 35.14, SD = 8.61). 73.3% of the participants were women, 26% were men, and 0.8% identified as other (self-identified as fluid and non-binary). Regarding this variable, according to data provided by the Chilean Ministry of Education, over 70% of teachers are women. Therefore, we consider the sample to be reasonably representative of the national context in terms of gender (CPEIP, 2020). Additionally, 16.8% of participants identified as Indigenous, primarily Mapuche. It should be noted that the intercultural construct does not refer to a specific culture, but rather to the interaction between different cultures based on dialogue, mutual respect, and recognition (Pereda, 2004). In this sense, the instrument (Intercultural Effectiveness Scale) is not specifically adapted for indigenous cultures. The teachers came from various educational institutions: public (46.6%), subsidized (37.4%), and private (16%), with an average of 9.25 years of professional experience (SD = 7.37).

### 2.2 Instruments

Data collection was conducted through a single survey consisting of three parts. Part one comprised the sociodemographic variables age, sex, ethnicity, educational institution, and years of teaching experience. In Part 2, the Spanish adaptation of the IES (Beltrán-Véliz et al., 2024) (Appendix A) was employed. This scale measures intercultural effectiveness, understood as the behavioral construct of intercultural communication. The IES is answered using an ordinal response scale (1 = strongly disagree, 5 = strongly agree) and is composed of 20 items distributed across six factors: behavioral flexibility (4 items), interaction relaxation (5 items), interactant respect (3 items), message skills (3 items), identity maintenance (3 items), and interaction management (2 items). Although item 18 in the validation study by Beltrán-Véliz et al. (2024) showed an insufficient factor loading, it was retained from the original version (Portalla and Chen, 2010) to evaluate its performance in a different sample. In this psychometric study conducted in Chile, Beltrán-Véliz et al. (2024) reported a good fit for the six-factor correlated model (MLR-*χ*^2^ (df = 155) = 488.297; *p* < 0.01; RMSEA = 0.058 [C.I. = 0.052–0.063]; CFI = 0.923; TLI = 0.906). In part 3, as a convergent validity measure, the MAS (Rozaimie et al., 2011) was applied. This instrument consists of nine items distributed in the following three factors: implicit culture awareness (4 items, i.e., ‘I accept and respect that male-female roles in families may vary significantly among different cultures.), tacit culture awareness (2 ítems, i.e. ‘It is important to identify immediately the ethnic groups of a person we meet or communicate with’), and cultural interaction awareness (3 ítems, i.e. ‘I discourage people from using racial and ethnic slurs or insult statement or behavior.’). The use of the MAS as a measure of convergent validity is theoretically justified, as both instruments share a conceptual foundation related to intercultural competence. While the IES focuses on behavioral skills, the MAS addresses the development of awareness of cultural diversity as the basis for such interactions. The MAS scale demonstrated acceptable internal consistency, as measured by Cronbach’s alpha, with a value of 0.717 (Rozaimie et al., 2011).

### 2.3 Procedure

A language adaptation procedure was conducted, and the original scales were translated into Spanish, with the Chilean language context taken into account. This procedure followed the ITC Guidelines for Translating and Adapting Tests (International Test Commission, 2017). To ensure linguistic equivalence, a three-stage adaptation procedure was employed. First, forward translations were conducted by two independent bilingual experts; second, the back-translation phase was conducted by two native English-speaking academics; third, to refine the instrument and eliminate potential comprehension errors, monolingual speakers performed a final review of the items. The process culminated in a consensus between researchers and translators to finalize the scale. English teachers from across Chile were invited to participate in the study through a combination of purposive and snowball sampling methods. Initially, email invitations were distributed to potential participants using the principal investigator’s university’s alumni databases. These databases included contact information for graduates from education and English language teaching programs, many of whom are currently working as in-service teachers throughout the country. To expand the participant pool, the snowball sampling technique was employed (Parker et al., 2019). Teachers who received the invitation were encouraged to share it with colleagues who met the inclusion criteria, specifically those currently working as in-service English teachers in Chile. Additionally, the invitation and survey link were posted and circulated through dedicated social media groups and pages created specifically for the study, targeting English teachers in Chile. All participants provided informed consent via an online form approved by the Ethics Committee of the sponsoring university before completing the survey.

### 2.4 Data analysis

Firstly, descriptive statistics of the scale were analyzed to estimate measures of central tendency, dispersion, and shape. In addition, univariate normality assessments were conducted for the 20 items of the scale using SPSS v.25. To evaluate the theoretical structure of the scale, confirmatory factor analyses (CFAs) were conducted using Mplus v.8.1 (Muthén and Muthén, 2017). For its implementation, the polychoric correlations matrix and the unweighted least squares with mean and variance adjustment (ULSMV) estimation method were used. Regarding Mardia’s test, significant deviations from multivariate normality were observed, both in skewness (Skewness = 79.5, *χ*^2^ = 5,170, *p* < 0.001) and kurtosis (Kurtosis = 561.3, *z* = 40.4, *p* < 0.001). Therefore, the data do not meet the assumption of multivariate normality (Mardia, 1970). To assess model quality, several goodness-of-fit indices were used to evaluate the CFA models: ULSMV-*χ*^2^, comparative fit index (CFI), Tucker–Lewis index (TLI), and root mean square error of approximation (RMSEA). For CFI and TLI, values of 0.90 or higher were considered acceptable (Schumacker and Lomax, 2016). For RMSEA, values less than or equal to 0.080 were considered a reasonable fit (Browne and Cudeck, 1992). To assess convergent validity, Pearson’s r correlation coefficient was used, following the criteria established by Cohen (2013) and Morales et al. (2003). To estimate reliability, McDonald’s *ω* was used as the primary reliability estimator, given its appropriateness for CFA-based models, while Cronbach’s *α* was reported as a complementary index (Green and Yang, 2015; Trizano-Hermosilla and Alvarado, 2016). Values of 0.7 or greater were considered satisfactory indicators of reliability, according to the criteria of Nunnally and Bernstein (1994).

## 3 Results

### 3.1 Descriptive analysis

Table 1 shows the descriptive results of the items. The highest mean is obtained by item 15, “I always show respect for my interlocutors during interaction with people from different cultures”. In contrast, the item with the lowest mean is item 6, “I have grammatical problems when interacting with people from different cultures”. Likewise, the univariate normality of the scale items was assessed. As shown in Table 1, none of the items complies with the univariate normality assumption, as they deviate significantly from what is expected under a normal distribution.

**Table 1:** Descriptive statistics.

Items	Mean	Standard deviation	Asymmetry	Kurtosis	K–S test
1. I find it easy to talk with people from different cultures.	4.01	0.961	−1.285	1.825	0.302
2. I am afraid to express myself when interacting with people from different cultures.	4.08	0.901	−0.916	0.506	0.258
3. I find it easy to get along with people from diverse cultural backgrounds.	4.10	0.862	−1.138	1.706	0.282
4. I am not always the person I appear to be when interacting with people from different cultures.	3.70	1.066	−0.575	−0.355	0.237
5. I am able to express my ideas clearly when interacting with people from different cultures.	4.05	0.721	−0.736	1.436	0.304
6. I have problems with grammar when interacting with people from different cultures.	3.32	1.022	−0.212	−0.703	0.225
7. I am able to answer questions effectively when interacting with people from different cultures.	4.10	0.694	−0.600	0.709	0.299
8. I find it difficult to feel my culturally different counterparts are similar to me.	3.97	0.951	−0.832	0.343	0.252
9. I use appropriate eye contact when interacting with people from different cultures.	4.34	0.699	−1.224	3.087	0.272
10. I have problems distinguishing between informative and persuasive messages when interacting with people from different cultures.	3.75	0.885	−0.453	−0.127	0.262
11. I always know how to initiate a conversation when interacting with people from different cultures.	3.54	0.922	−0.336	−0.252	0.241
12. I often miss parts of what is going on when interacting with people from different cultures.	3.62	0.921	−0.567	−0.143	0.296
13. I feel relaxed when interacting with people from different cultures.	3.78	0.825	−0.323	−0.235	0.263
14. I often act like a very different person when interacting with people from different cultures.	3.98	0.935	−0.880	0.377	0.278
15. I always show respect for my culturally different counterparts during our interaction.	4.65	0.565	−2.172	8.588	0.412
16. I always feel a sense of distance with my culturally different counterparts during our interaction.	3.99	0.891	−0.851	0.688	0.27
17. I find I have a lot in common with my culturally different counterparts during our interaction.	3.65	0.784	0.103	−0.390	0.251
18. I find the best way to act is to be myself when interacting with people from different cultures.	4.39	0.840	−1.563	2.556	0.326
19. I find it easy to identify with my culturally different counterparts during our interaction.	3.72	0.959	−0.548	0.131	0.232
20. I always show respect for the opinions of my culturally different counterparts during our interaction.	4.62	0.556	−1.277	1.669	0.402

K–S test, Kolmogorov–Smirnov test.

### 3.2 Confirmatory factor analysis

**Figure 1:**
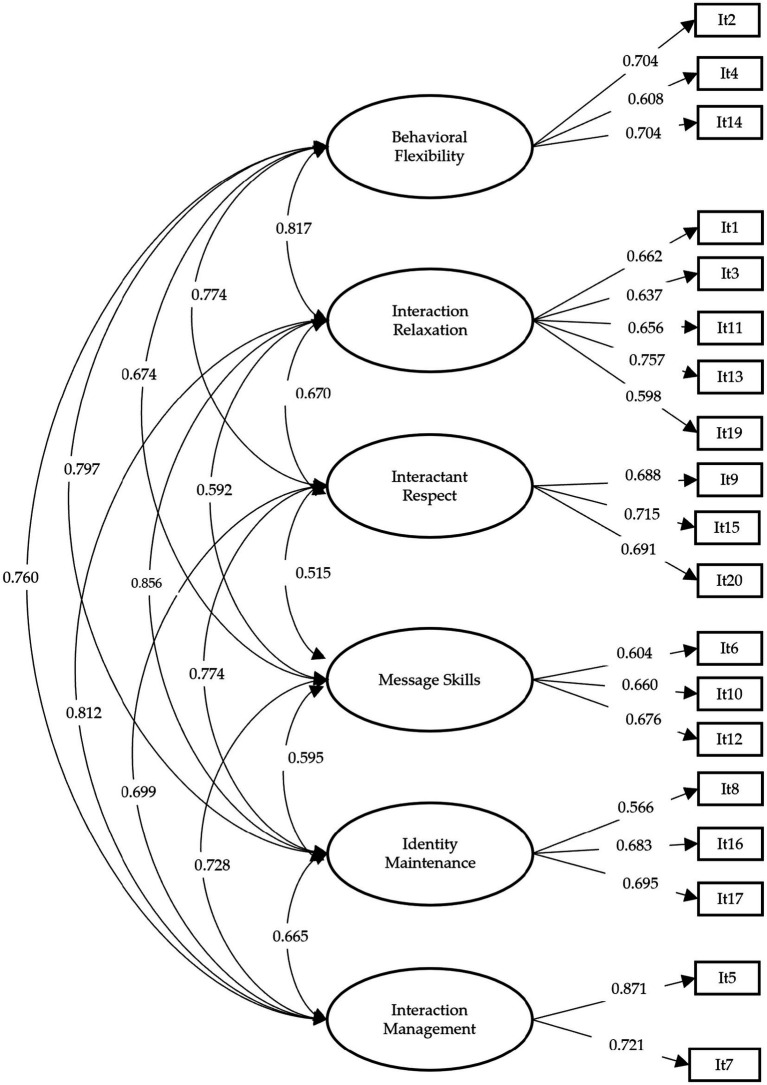
Six-factor model of the confirmatory factor solution. The figure shows standardized values.

A confirmatory factor analysis was performed to evaluate the structure of the IES (Figure 1), which was based on the scale’s theoretical proposal of six correlated factors. The results showed appropriate goodness-of-fit indices (ULSMV-*χ*^2^ (gl = 155) = 460.458; *p* < 0.01; RMSEA = 0.077 [C.I. = 0.069–0.085]; CFI = 0.929; TLI = 0.913; SRMR = 0.054). Considering these results, the IES confirms its factorial structure of six correlated factors in a population of Chilean EFL in-service teachers.

### 3.3 Correlational analysis

After confirming the factorial structure of the IES, its convergent validity was examined by correlating the IES factors with the three MAS factors (Table 2). Overall, the results indicate that several IES factors are positively associated with Implicit Culture Awareness (F7). This suggests that dimensions such as Behavioral Flexibility, Interaction Management, Interactant Respect, and Identity Maintenance are related to more implicit forms of cultural awareness. Significant associations were also found with Cultural Interaction Awareness (F8), particularly in factors related to interaction and adaptation, which supports the idea that intercultural sensitivity is expressed in both explicit and implicit ways. Finally, the consistent relationships between various IES factors and Tacit Culture Awareness (F9) demonstrate that the scale also connects with deeper, less conscious components of the intercultural experience. To sum up, this pattern of correlations confirms the convergent validity of the IES, indicating that its factors align coherently with the three MAS dimensions, which reflect different levels of cultural awareness in intercultural interactions.

**Table 2:** Correlational analysis between the IES and MAS scales.

Factors	F1	F2	F3	F4	F5	F6	F7	F8	F9
F1	1								
F2	0.492^**^	1							
F3	0.411^**^	0.380^**^	1						
F4	0.431^**^	0.333^**^	0.266^**^	1					
F5	0.462^**^	0.513^**^	0.417^**^	0.359^**^	1				
F6	0.469^**^	0.529^**^	0.452^**^	0.473^**^	0.400^**^	1			
F7	0.160^**^	0.133^**^	0.332^**^	0.077 ns	0.194^**^	0.168^**^	1		
F8	0.028 ns	0.128^*^	0.194^**^	−0.019 ns	0.072 ns	0.076 ns	0.274^**^	1	
F9	0.031 ns	0.192^**^	0.176^**^	−0.005 ns	0.033 ns	0.117^*^	0.321^**^	0.274^**^	1

^**^p < 0.01; ^*^p < 0.05. F1, Behavioral Flexibility; F2, Interaction Relaxation; F3, Interactant Respect; F4, Message Skills; F5, Identity Maintenance; F6, Interaction Management; F7, Implicit culture awareness; F8, Cultural interaction awareness; F9, Tacit culture awareness.

### 3.4 Reliability

Based on the validity evidence, reliability was primarily evaluated using McDonald’s *ω* coefficients. As shown in Table 3, the scale exhibits generally acceptable reliability values according to *ω*, with some variability across factors. The Interaction Relaxation factor had the highest value, while the Message Skills had the lowest. These results show that the IES instrument presents an adequate value for its application in the Chilean teaching population.

**Table 3:** Reliability analysis.

Factors	McDonald’s *ω*	Cronbach’s *α*
Behavioral flexibility	0.704	0.691
Interaction relaxation	0.723	0.719
Interactant respect	0.647	0.595
Message skills	0.635	0.634
Identity maintenance	0.608	0.606
Interaction management	0.684	0.684

It should be noted that the Interaction Management factor comprises only two items. Therefore, the reliability estimates for this factor should be interpreted with caution, as internal consistency coefficients such as Cronbach’s *α* and McDonald’s *ω* are sensitive to the number of items. To enhance transparency, the inter-item correlation between the two items was examined and showed a moderate positive association, supporting an acceptable level of consistency for a two-item factor.

The results of this study reinforce the applicability of the IES for evaluating and fostering intercultural effectiveness in EFL teachers. First, the descriptive analysis highlights the variability of the items, with the highest scores relating to respect for intercultural interlocutors and the lowest reflecting difficulties with perceived grammatical accuracy. Second, confirmatory factor analysis confirms the six-factor structure of the IES, with acceptable fit indices aligning with theoretical expectations. Third, the correlational analysis demonstrates meaningful, statistically significant relationships between the IES factors and the implicit culture awareness factor of the MAS scale, further supporting convergent validity. Finally, reliability estimates, though variable, suggest a differential utility of the subscales, with some factors showing adequate consistency and others presenting limitations that should be considered when interpreting factor-level scores.

## 4 Discussion

This study aimed to validate the Spanish version of the IES in a sample of in-service Chilean EFL teachers. The findings confirm the reliability and factorial validity of the IES, aligning with previous studies that have utilized this instrument across diverse cultural contexts. The results confirm the fulfillment of the two initially proposed hypotheses: (H1) the scores of the IES will meet adequate levels of validity and reliability in the sample of in-service Chilean EFL teachers, and (H2) the scores of the six factors of the IES will present positive and statistically significant correlations with the three factors of the MAS scale.

The confirmatory factor analysis supported the six-factor model proposed by Portalla and Chen (2010) and yielded acceptable goodness-of-fit indices. These results align with prior validations conducted in different cultural contexts, such as Turkey and others (Arslan et al., 2015; Yakar and Alpar, 2017; Yilmaz et al., 2020; Beltrán-Véliz et al., 2024). This consistency proves the IES’s adaptability and theoretical consistency across diverse populations. In the Chilean sample, all six factors—Behavioral Flexibility, Interaction Relaxation, Interactant Respect, Message Skills, Identity Maintenance, and Interaction Management – contributed significantly to the overall construct, confirming the scale’s structural integrity in this new cultural setting. These results suggest that the IES is a robust tool for assessing intercultural effectiveness in a Chilean educational setting, where cultural diversity and the need for intercultural communication competencies are increasingly recognized (CPEIP, 2021). Moreover, the reliability estimates for each factor, while varied, were within acceptable ranges, reinforcing the scale’s applicability. This validation might be useful for other Spanish-speaking countries that have decided to integrate ICC into their EFL teaching approaches (Álvarez, 2020; Hernández-López et al., 2024). However, it would be necessary to consider fither cultural specificity for other contexts, as established by the ITC Guidelines for Translating and Adapting Tests (International Test Commission, 2017). In this regard, cultural meanings should be further explored. For instance, in collectivist cultures, behavioral flexibility might be prized as cultural intelligence (Bernardo and Presbitero, 2018), where adapting one’s demeanor to fit the hierarchy is a sign of respect and education. Conversely, identity maintenance—the drive to remain consistent across all contexts— might often be viewed as “rigidity,” as it prioritizes individual ego over collective harmony. In these societies, the “self” is not a fixed monument to be defended, but a dynamic tool used to maintain social order and preserve group cohesion. This creates tension between the Identity Maintenance and Behavioral Flexibility factors, as answers will depend on cultural beliefs and values.

Another aspect that might benefit from further research is social desirability. The high mean score on the Interactant Respect subscale (*M* = 4.65) might indicate the presence of Social Desirability Bias (SDB). For instance, in the Chilean context, teachers may feel a “normative obligation” to report high respect to preserve group harmony in an intercultural setting, suggesting an idealized competence rather than real classroom behavior. Future research using the IES would benefit from incorporating a Social Desirability Scale and English Language Anxiety metrics, in order to distinguish between participants’ actual intercultural skills and the effects of social masking or linguistic apprehensions.

The correlations between the IES factors and factors of the Multicultural Awareness Scale (MAS) provided evidence of convergent validity. Positive correlations were observed between the Implicit Culture Awareness and the factors Interactant Respect (*ρ* = 0.332, *p* < 0.01) and Identity Maintenance (*ρ* = 0.194, *p* < 0.01), showing moderate relationships. The Cultural Interaction Awareness factor correlated moderately with the Interaction Relaxation factor (*ρ* = 0.128, *p* < 0.01) and the Interactant Respect factor (*ρ* = 0.194, *p* < 0.01). The Tacit Culture Awareness factor correlated moderately with the Interaction Relaxation factor (*ρ* = 0.192, *p* < 0.01) and the Interactant Respect factor (*ρ* = 0.196, *p* < 0.01). These findings align with theoretical expectations, highlighting the interconnectedness of behavioral and cognitive dimensions in fostering ICC.

Regarding reliability, the study reveals a mixed but generally acceptable profile for the Intercultural Effectiveness Scale (IES). Regarding reliability, the study reveals a mixed but generally acceptable profile for the Intercultural Effectiveness Scale (IES) when considering McDonald’s *ω* coefficients. The Interaction Relaxation factor demonstrated the highest reliability (*ω* = 0.723), while Message Skills, Interactant Respect, and Identity Maintenance showed comparatively lower *ω* values. Although these coefficients are close to the acceptable threshold, they suggest a more cautious interpretation of these subscales and indicate potential areas for future refinement. It should be noted that Message Skills, Interactant Respect, and Identity Maintenance showed lower values. In this regard, the Identity Maintenance factor included one item (Item 18) with a relatively low factor loading. As a test of robustness, we examined whether removing this item would significantly improve the model fit. This post-hoc inspection did not reveal any substantial changes in the fit indices, supporting the decision to retain the item for theoretical reasons. This variability suggests that, while the overall instrument is sufficiently consistent for its intended use with Chilean teachers, the items in these specific, less reliable subscales may not capture their intended concepts with the same degree of precision, warranting a more cautious interpretation of their individual scores. Therefore, subscale scores should be interpreted with caution when reliability indices approach the minimum acceptable threshold (approximately *ω* = 0.60), especially when results are used for interpretive or applied purposes.

The validation of the IES for in-service EFL teachers in Chile underscores the instrument’s utility for professional development programs. Given the increasing emphasis on fostering ICC in teacher training curricula (CPEIP, 2021), the IES can serve as a tool for program evaluation (aggregate data) or self-reflection. Teachers with higher levels of ICC are better positioned to create inclusive classroom environments, model effective intercultural communication strategies, and prepare students for global citizenship (Byram et al., 2001). The findings also have implications for addressing gaps in teacher education. For example, the relatively lower reliability scores for certain factors may indicate areas where teachers require additional support, such as developing grammatical accuracy and distinguishing between different types of messages in intercultural interactions. These areas could be targeted through tailored workshops and continuous professional development initiatives. The moderate scores on Interaction Management suggest potential areas of improvement for Chilean EFL teachers, echoing challenges identified in similar educational contexts (Bates and Rehal, 2017).

Among the study’s limitations, the use of a non-probabilistic sampling method may limit the generalizability of the findings. Additionally, while the IES has demonstrated reliability and validity, further research is needed to examine its predictive validity with respect to specific educational outcomes in the teaching-learning environment, and to explore how ICC might relate to other variables, such as school climate. Future studies should also investigate the role of demographic variables, such as ethnicity, teaching experience, and type of exposure to intercultural relations, in shaping intercultural effectiveness throughout teachers’ lives. Given the multicultural composition of Chilean classrooms, understanding these dynamics could offer deeper insights into how teachers’ intercultural communication competencies shape their pedagogical practices. Additionally, longitudinal studies examining the development of intercultural effectiveness over time would be valuable. Such research could assess the impact of targeted interventions, such as intercultural training programs, on the professional growth of EFL teachers. Furthermore, future research should incorporate samples with greater gender balance, which would allow for the evaluation of measurement invariance and, once scalar invariance has been established, explore possible gender differences through comparisons of latent means. Also, future research should examine the divergent validity of the instrument in order to provide additional evidence of its differentiation from conceptually related constructs and thus strengthen the psychometric soundness and structural interpretation of the model.

To conclude, this study validates the Spanish adaptation of the IES for its use with in-service Chilean EFL teachers. The findings highlight the scale’s reliability and factorial validity and generally acceptable reliability, as evidenced primarily by McDonald’s *ω* coefficients, supporting its potential as a tool in program evaluation and self-reflection. Overall, positive and statistically significant correlations were observed between the IES and the MAS for several, but not all, factors. In particular, evidence of convergent validity was supported for multiple IES dimensions in relation to the MAS, whereas the Message Skills factor did not show significant correlations with any MAS dimension. This suggests the need for further research on non-native English teachers.

The results obtained for the ‘Message Skills’ factor showed a lack of evidence for convergent validity with the MAS factors, suggesting that this dimension may be influenced by additional sources of variance. This may be driven by confounding variables such as general language confidence, communication anxiety (Akkakoson, 2016), and professional linguistic anxiety (Gargalianou et al., 2016). These influences are particularly evident in Item 6 (“I have problems with grammar when interacting with people from different cultures”), which captures linguistic insecurities that extend beyond purely intercultural barriers. Message Skills require real-time linguistic production, which, for Non-Native Speaker (NNS) teachers, is often mediated by their Affective Filter (Lin, 2008). If the assessment reflects a teacher’s situational stress rather than their actual communicative competence, it introduces construct-irrelevant variance, thereby weakening the tool’s validity. Consequently, Message Skills may function less as a measure of linguistic proficiency and more as a proxy for the psychological comfort of the educator, which might change based on the perceived scrutiny of the evaluator.

By addressing areas for growth and aligning professional development initiatives with the demands of a globalized educational context, the IES can foster more inclusive and effective teaching practices that enable understanding of various intercultural aspects and, therefore, effective communication.

Future research should continue in different areas of ICC evaluation. On the one hand, to explore the interplay between ICC and educational outcomes in the EFL context, ensuring that teacher training programs remain responsive to the evolving needs of diverse classrooms; on the other, ensuring that the adaptations of different instruments that assess this competence comprise not only the linguistic but also the cultural aspects, which constitutes one limitation of this study, considering the IES was validated for the Chilean Spanish-speaking context. In this respect, it would be necessary to test whether this instrument accounts for other cultural contexts in which Spanish is also spoken, as problematized by the International Test Commission (2017) regarding the importance of considering culture when adapting instruments.

Additionally, building on the current validation of the Intercultural Effectiveness Scale using the Multicultural Awareness Scale (MAS), future research should expand the nomological network to include additional constructs that capture the broader dimensions of intercultural communication. In particular, incorporating measures such as the Intercultural Sensitivity Scale (Chen and Starosta, 2000) would allow for a more comprehensive assessment of the affective domain and establish convergent validity across different facets of intercultural competence. Moreover, future studies should include discriminant constructs such as modern racism, ethnocentrism, or social dominance orientation to ensure the scale does not overlap with unrelated or undesirable traits. Longitudinal or cross-cultural research designs could also provide insight into the scale’s predictive validity and cultural invariance across diverse populations. Finally, replicating the validation with larger and more diverse samples, including in-service professionals and pre-service educators in intercultural contexts, would strengthen the generalizability of the findings and support the scale’s use in both educational and organizational settings.

## Data Availability

The dataset for this study is available from the corresponding author upon reasonable request due to ethical restrictions.
