# Supplementary material for: Psychometric properties of the intercultural effectiveness scale in a sample of in-service Chilean EFL teachers
Source: Front Psychol. 2026 Feb 17;17:1734563. doi: 10.3389/fpsyg.2026.1734563 (PMC12953132; doi:10.3389/fpsyg.2026.1734563)
Supplement: Supplementary file 1 [file Supplementary_file_1.docx]

Supplementary Material / Material suplementario

# Spanish version of the IES

# Escala de Efectividad Cultural:

A continuación, se presentan una serie de afirmaciones. Por favor, seleccione rápidamente su primera impresión respecto de su nivel de acuerdo o desacuerdo para cada afirmación. Marque sólo una respuesta por enunciado. No hay respuestas correctas o incorrectas

Escriba el número correspondiente a su respuesta en el espacio en blanco que aparece antes de la afirmación:

1._______ Se me hace fácil comunicarme con personas de culturas diferentes.

2._______ Tengo miedo de expresarme cuando interactúo con personas de culturas

diferentes.

3._______ Se me hace fácil llevarme bien con personas de culturas diferentes.

4._______ No soy siempre la persona que parezco ser cuando interactúo con personas

de culturas diferentes.

5._______ Puedo expresar claramente mis ideas cuando interactúo con personas de

culturas diferentes.

6._______ Tengo problemas gramaticales cuando interactúo con personas de culturas

diferentes.

7._______ Puedo responder preguntas eficazmente cuando interactúo con personas

de culturas diferentes.

8._______ Se me hace difícil sentir que las personas de otras culturas con las que

interactúo son similares a mí.

9._______ Uso un contacto visual apropiado cuando interactúo con personas de

culturas diferentes.

10._______ Tengo problemas en distinguir mensajes informativos de mensajes

persuasivos cuando interactúo con personas de culturas diferentes.

11._______ Siempre sé cómo comenzar una conversación cuando interactúo con

personas de culturas diferentes.

12._______ A menudo no comprendo algunas partes de lo que está ocurriendo

cuando interactúo con personas de culturas diferentes.

13._______ Me siento relajado cuando interactúo con personas de culturas diferentes.

14._______ A menudo actúo como una persona muy distinta cuando interactúo con

personas de culturas diferentes.

15._______ Siempre muestro respeto por mis interlocutores durante la interacción

con personas de culturas diferentes.

16._______ Siempre tengo un sentimiento de distancia con mis interlocutores durante

la interacción con personas de culturas diferentes.

17._______ Creo que tengo mucho en común con mis interlocutores durante la

interacción con personas de culturas diferentes.

18._______ Creo que la mejor manera de comportarme es ser yo mismo cuando

interactúo con personas de culturas diferentes.

19._______ Se me hace fácil identificarme con mis interlocutores durante la

interacción con personas de culturas diferentes.

20._______ Siempre muestro respeto por las opiniones de con mis interlocutores

durante la interacción con personas de culturas diferentes.

Note. Items 2, 4, 6, 8, 10, 12, 14, 16, and 18 should be reverse-coded before summing the 20

items. Behavioural Flexibility items are 2, 4, 14, and 18; Interaction Relaxation items are 1, 3, 11,

13, and 19; Interactant Respect items are 9, 15, and 20; Message Skills items are 6, 10, and 12;

Identity Maintenance items are 8, 16, and 17; Interaction Management items are 5 and 7.
